# Decreased *TRPM7* inhibits activities and induces apoptosis of bladder cancer cells via ERK1/2 pathway

**DOI:** 10.18632/oncotarget.12146

**Published:** 2016-09-20

**Authors:** Rui Cao, Zhe Meng, Tongzu Liu, Gang Wang, Guofeng Qian, Tingting Cao, Xinyuan Guan, Hancai Dan, Yu Xiao, Xinghuan Wang

**Affiliations:** ^1^ Department of Urology, Zhongnan Hospital of Wuhan University, Wuhan, China; ^2^ Department of Endocrinology, The First Affiliated Hospital of Zhejiang University, Hangzhou, China; ^3^ Department of Clinical Oncology, Li Ka Shing Faculty of Medicine, University of Hong Kong, Hong Kong; ^4^ Greenebaum Cancer Center, School of Medicine, University of Maryland, Baltimore, MD, USA; ^5^ Center for Medical Science Research, Zhongnan Hospital of Wuhan University, Wuhan, China

**Keywords:** TRPM7, bladder cancer, apoptosis, MAPK, AKT

## Abstract

Transient receptor potential melastatin 7 (TRPM7) functions as a Mg^2+^/Ca^2+^-permeable channel fused with a kinase domain and regulates various physical processes and diseases. However, its effects on pathogenesis of human bladder cancer (BCa) has not been clarified yet. Our microarray analysis has suggested that calcium signaling pathway is connected with bladder cancer via MAPK pathway. Therefore, we aim to investigate the mechanism of TRPM7 in BCa tumorigenesis by using BCa tissues compared with normal bladder epithelium tissues, as well as using distinct BCa cell lines (EJ, 5637 and T24). We observed increased *TRPM7* expression and dysregulation of proteins involved in Epithelial-Mesenchymal Transition (EMT) in BCa tissues. Moreover, knockdown of *TRPM7* in BCa cells reversed the EMT status, accompanied by increase of reactive oxygen species (ROS). Furthermore, *TRPM7* deficiency could inhibit BCa cell proliferation, migration and invasion, as well as induce p-ERK1/2 and suppress PI3K/AKT at the protein level. Downregulation of *TRPM7* promoted cell cycle arrest at G0/G1 phase and apoptosis *in vitro*, which could be recovered by pre-treatment with U0126 to deactivate ERK1/2, suggesting a close correlation between TRPM7 and the MAPK signaling pathway. Furthermore, a NOD/SCID mouse model transplanted using the BCa cells was established, revealing delayed tumor growth by reduced protein activity and mRNA transcription of *TRPM7 in vivo*. Our results suggested *TRPM7* might be essential for BCa tumorigenesis by interfering BCa cell proliferation, motility and apoptosis.

## INTRODUCTION

Bladder cancer (BCa) is one of the most common cancers worldwide [1]. Despite recent progress, the molecular mechanism underlying BCa pathogenesis remains to be further elucidated. Therefore, our group has generated a microarray analysis using total RNA isolated from several bladder cancer tissues comparing with normal bladder epithelium [2], suggesting calcium signaling pathway was linked with bladder cancer via MAPK signaling pathway, which was connected with cell cycle.

Mitogen-activated protein kinases (MAPKs) play a key role in signal transduction from cell membrane to nucleus in response to a wide range of stimuli and are involved in the regulation of cell proliferation [3], survival, differentiation [4] and apoptosis [5]. Importantly, aberrant regulation of MAPK could contribute to cancer and other human diseases, including bladder cancer [6, 7]. Many studies have suggested that intracellular or extracellular calcium disorders might induce abnormal activation or deactivation of MAPK cascades [8, 9] and consequently initiate cancer development [10]. A recent publication using pathway network analyses revealed a major overlaps with various diseases and convergence upon MAPK and calcium signaling as well [11]. Studies using U0126, a selective MAPK kinase (MKK) inhibitor [12], have revealed a major effect on the deactivation of ERK1/2 [13] possibly even affecting MAPK-mediated mitochondrial-derived [14] and endoplasmic reticulum stress induced apoptosis [15].

Transient receptor potential melastatin 7 (TRPM7) is a member of “chanzymes”, which function as a Mg^2+^/Ca^2+^-permeable channel fused with a kinase. TRPM7 has been reported to be ubiquitously expressed in various human tissues [16–18], suggesting that it could be implicated in important physiological processes such as cellular Mg^2+^ homeostasis [18], cell viability and growth [19], anoxic neuronal cell death [20], and cell adhesion [21]. Recent studies indicated a close correlation between TRPM7 and cancer, demonstrating its involvement in retinoblastoma [22], gastric cancer [23], breast cancer [24–27], nasopharyngeal carcinoma [28], pancreatic cancer [29], prostate cancer [30], and ovarian carcinoma [31]. Our previous studies have suggested that knockdown of *TRPM8*, another important subtype of the TRPM family, could inhibit proliferation of osteosarcoma and prostate cancer cells [32, 33]. In addition, our studies have suggested that TRPM7 affects kidney injury [34], revealing a correlation between the TRPM family and cell ability [35], cell growth as well as Epithelial-Mesenchymal Transition (EMT) [36], which was involved in malignancy of tumor. Another publication has reported that during stress condition, such as brain ischaemia, TRPM7 could control the level of reactive oxygen species (ROS) [37], which could have a Cross Talk with the EMT [38]. TRPM7 has been reported to be expressed in human and mouse urothelium [39, 40], as well as in MBT-2 mouse bladder cancer cells and T24 human bladder cancer cells [41], but its effect and mechanism in human bladder cancer remain largely unknown. Therefore, we hypothesized that *TRPM7*, which is involved in the calcium signaling pathway, could affect bladder cancer by the MAPK signaling pathway to trigger BCa cell cycle arrest and apoptosis. We aim to identify the alteration of TRPM7 and related proteins involved in the EMT regulation using BCa tissues *in vivo*, and to observe its effects on EMT, cell migration/invasion, apoptosis and cell cycle in distinct BCa cell lines *in vitro* for a potential strategy of rescue experiment, as well as to analyze the influence of tumor growth using nude mice *in vivo* with deactivated TRPM7 and downregulated *TRPM7* at transcriptional level.

## RESULTS

### Microarray analysis revealed calcium and MAPK signaling pathways as central regulators in BCa development

**Figure 1:**
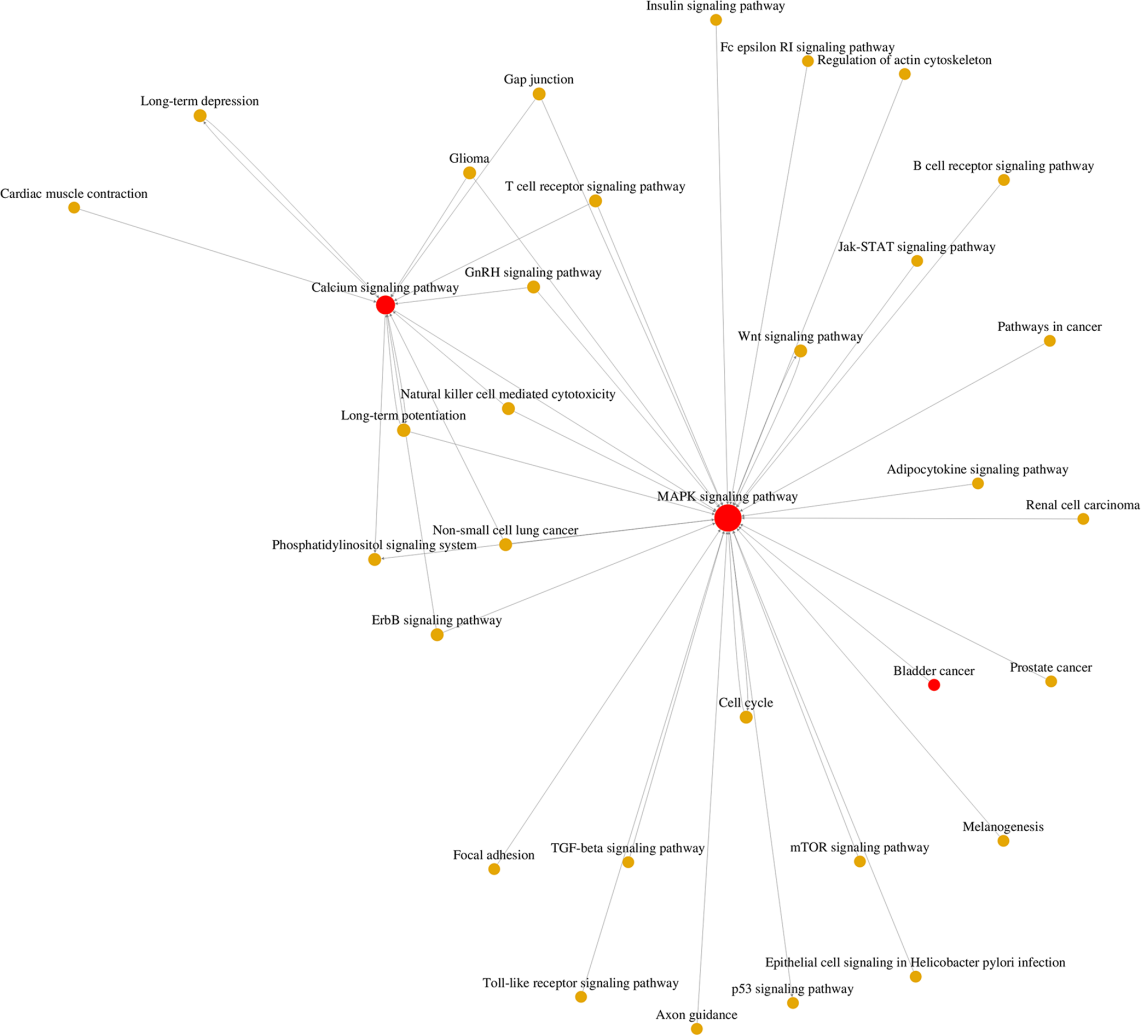
Microarray analysis using mRNA isolated from BCa tissues and normal bladder epithelium tissues From the microarray results, 1338 genes (fold change > 1.5, Supplementary Information 2) and 146 signaling pathways (Supplementary Information 3) were screened out. Gene ontology (GO) and Go-map network analysis by using the GCBI analysis tool suggested the calcium signaling pathway was at a central position associated with bladder cancer via the MAPK signaling pathway.

Three BCa tissues (stage II) and three normal bladder tissues were collected for alterations of mRNA by microarray analysis (Approval in Supplementary Information S1), suggesting 1338 genes (fold change > 1.5) (Supplementary Information S2) and 146 signaling pathways were significantly affected in the BCa tissues (Supplementary Information S3). Using a GCBI analysis tool, a pathway network connected to BCa was generated (Figure 1), indicating that a calcium signaling pathway was correlated with BCa via the MAPK signaling pathway connected with cell cycle regulation, as well as a central role of calcium and MAPK signaling pathways involved in the development of BCa. In addition, by annotation and overrepresentation analysis using our raw microarray data and DAVID database, we observed the genes involved in calcium signaling pathway were altered (Supplementary Figure S1A), accompanied by significantly upregulation of *CALM*, *CaN* and *CAMK* under the deficiency of *TRPM7 in vitro* (Supplementary Figure S1B–S1C). Therefore, we would like to investigate the alterations of the genes and proteins related with the pathways using bladder tissues and distinct BCa cell lines.

### Induction of *TRPM7* and dysregulation of EMT markers in BCa tissues

**Figure 2:**
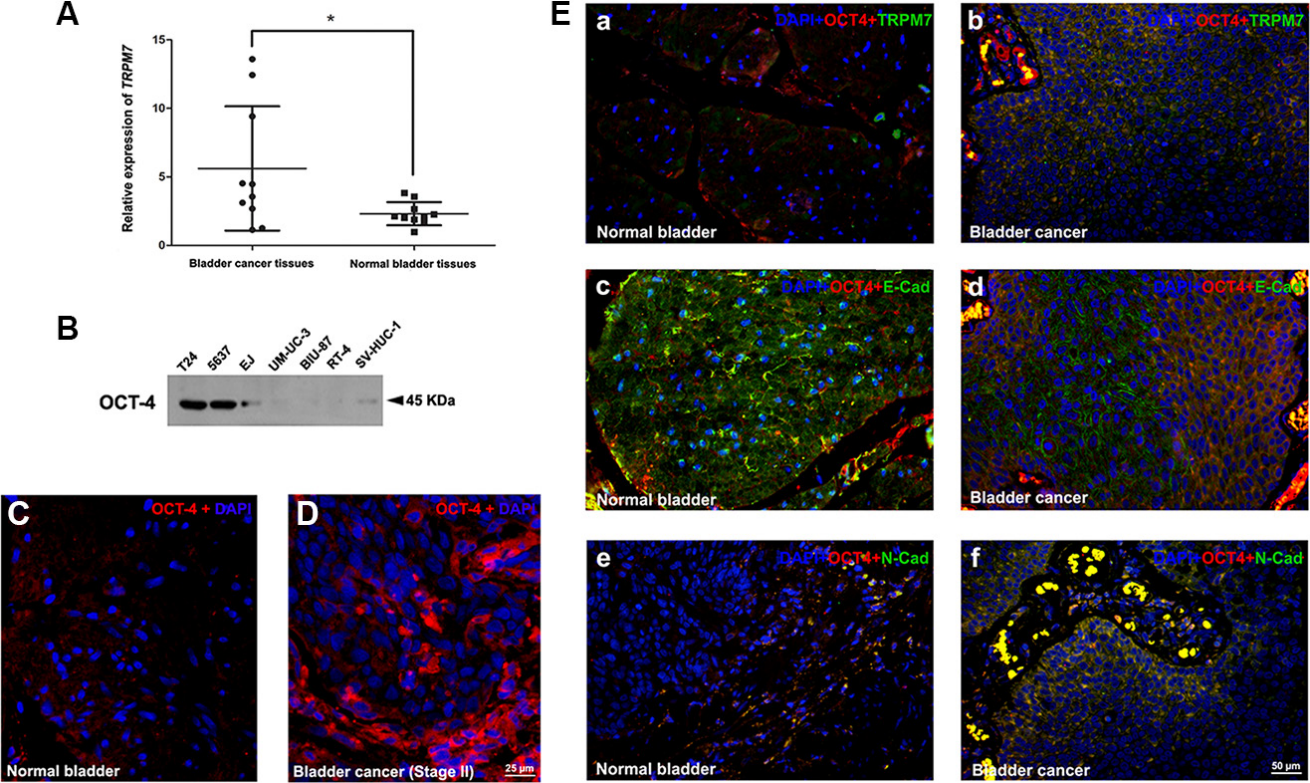
*TRPM7* is upregulated in the BCa tissues and correlated with EMT markers (**A**) qRT-PCR analysis of relative gene expression of TRPM7 in total RNA isolated from ten BCa tissues at stage II, comparing with ten normal bladder tissues. Significance of *TRPM7* expression difference was analyzed using *T-test*. *p < 0.05. (**B**) Western blot analysis of OCT-4 protein abundance in the human BCa cell lines (T24, 5637, EJ, UM-UC-3, BIU-87, RT-4) and immortalized normal uroepithelial cell line (SV-HUC-1), cell types and protein masses were indicated. (**C–D**) Representative immunofluorescence staining of OCT-4 (red) in the BCa tissue (D) comparing with the normal bladder tissue (C). Nuclears were stained by DAPI (blue). The images were photographed by fluorescence microscopy. The scale bar for C and D is 25 μm. (**E**) Representative double immunofluorescence staining of TRPM7, E-cadherin and N-cadherin (green) in the BCa tissues (b, d, f) comparing with normal bladder tissues (a, c, e). OCT-4 (red) was used as a marker of BCa cells, suggesting upregulation of TRPM7 and N-cadherin in the OCT-4 positive BCa cells (b and f), whereas a downregulation of E-cadherin (d). Nuclears were stained by DAPI (blue). The scale bar for E (a-f) is 50 μm.

Immunofluorescence staining using ten BCa tissues and ten normal bladder tissues revealed a strong increase of OCT-4 in the cytoplasmic region of the BCa tissues (representative staining in Figure 2C–2D). Distinct human BCa cell lines (from high malignancy to low malignancy: T24, 5637, EJ, UM-UC-3, BIU-87, RT-4) and immortalized normal uroepithelial cell line (SV-HUC-1) exhibited a downregulation tendency of OCT-4 by Western blot analysis (Figure 2B), suggesting OCT-4 could be a marker for bladder cancer. qRT-PCR revealed that transcription of *TRPM7* was upregulated in the BCa tissues compared with the normal bladder tissues (Figure 2A). TRPM7 was also induced in cytomembrane of the OCT4-positive cells in the BCa tissues (representative staining in Figure 2E a-b). Immunofluorescence analysis also suggested that distribution of proteins (E-cadherin and N-cadherin) involved in EMT process was strongly altered (representative staining in Figure 2E c-f). We observed a reduction of E-cadherin (Figure 2E c-d) and an increase of N-cadherin (Figure 2E e-f) in the OCT-4 positive cells in BCa tissues.

### Downregulation of *TRPM7* reversed dysregulation of EMT markers *in vitro*

**Figure 3:**
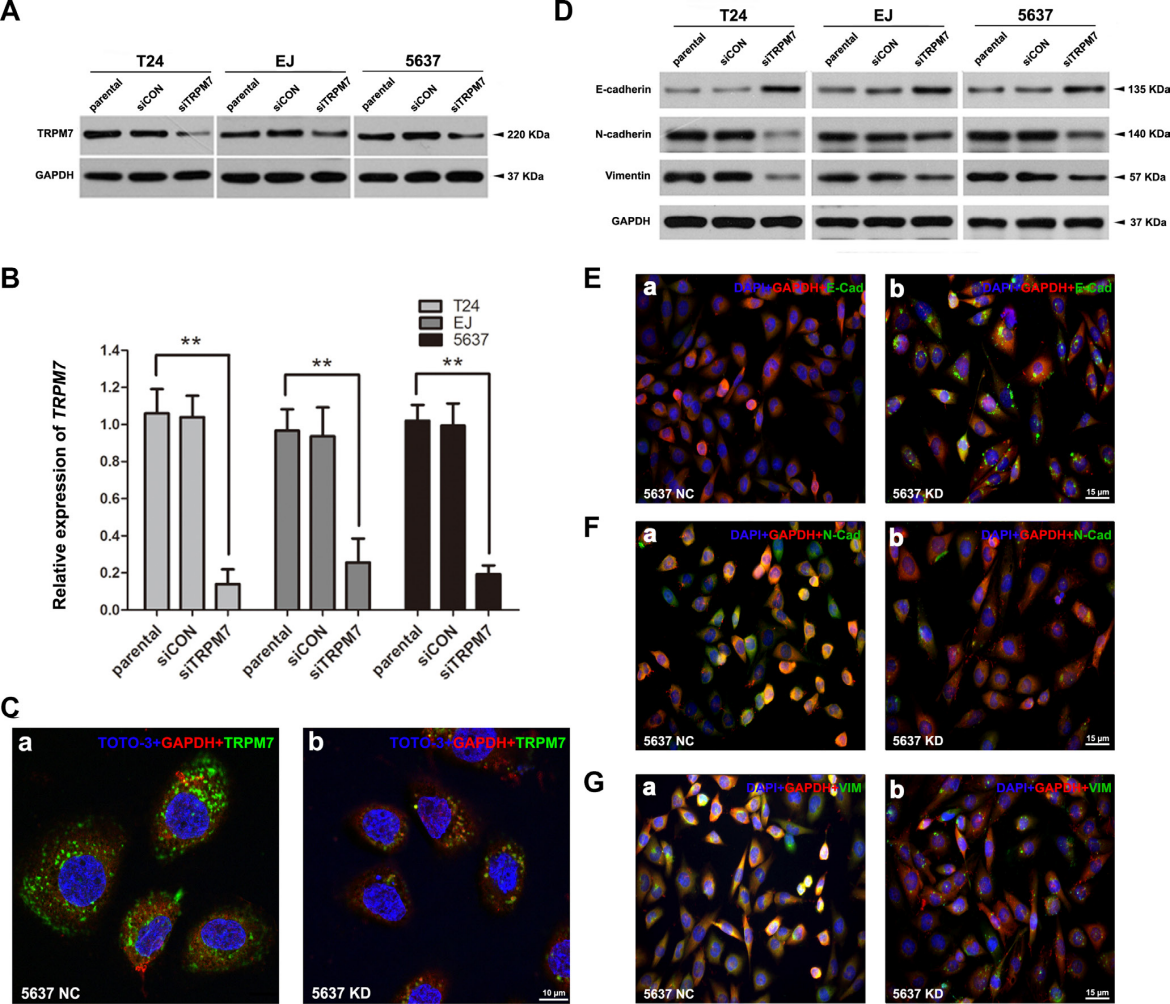
Knockdown of *TRPM7* reversed EMT status and alleviated malignancy in BCa cells (**A–C**) Efficiency of *TRPM7* knockdown by *siRNA* treatment in distinct BCa cells (T24, EJ, 5637) was verified by Western blot, qRT-PCR and double immunofluorescence staining analysis. (A) Western blot analysis revealed a strongly decreased protein abundance of TRPM7 by the *siTRPM7* treatment, comparing with parental and *siCON* treatment. GAPDH was used as a loading control (cell types, treatment of *siRNA* and protein masses were indicated). (B) The relative gene expression of *TRPM7* after *siRNA* treatment was analyzed by qRT-PCR, indicating a significantly downregulation of TRPM7 at gene expression level in the three BCa cells. Values shown were mean ± SD of triplicate measurements and repeated three times with similar results. Statistical significance was calculated using ANOVA. **p < 0.01. (C) Representative double immunofluorescence staining of TRPM7 (green) and GAPDH (red) in the 5637 cells after *siTRPM7* treatment (KD) (b), comparing with *siCON* treatment (NC) (a). Nuclears were stained by TOTO-3 (blue). The images were photographed by confocal microscopy. The scale bar for C (a-b) is 10 μm. The effect on EMT markers (E-cadherin, N-cadherin and Vimentin) in the BCa cells by *TRPM7* deficiency was investigated by Western blot and double immunofluorescence staining analysis. (**D**) Protein abundance of E-cadherin was increased by the *siTRPM7* treatment in the three cell lines, in contrast, a reduce of N-cadherin and Vimentin was noted by the Western blot analysis. GAPDH abundance was used as a control. Cell types, treatment of *siRNA* and protein masses were indicated. (**E–G**) Representative double immunofluorescence staining for E-cadherin (E), N-cadherin (F) and Vimentin (G) (green) in the 5637 cells after *siTRPM7* treatment (KD) (b) compared with *siCON* treatment (NC) (a). Nuclears were stained by DAPI (blue). The images were photographed by fluorescence microscopy. The scale bars for E-G are 15 μm.

To investigate the effects of TRPM7 in BCa, a model of *TRPM7* deficiency in distinct BCa cell lines (T24, EJ and 5637) was established by *siRNA* transfection. The knockdown efficiency of the *siRNA* was validated by qRT-PCR (Figure 3B), Western blot analysis (Figure 3A), and immunofluorescence staining (representative staining in Figure 3C), indicating *TRPM7* was remarkably silenced both at gene transcription and translation levels in the three BCa cells transfected by the *siRNA*. In contrast to the decrease of E-cadherin and increase of N-cadherin noticed in the BCa tissues (Figure 2E c-f). Knockdown of *TRPM7* triggered strong upregulation of the epithelial marker E-cadherin and considerable downregulation of the two mesenchymal markers N-cadherin and Vimentin, revealed by Western blot analysis (Figure 3D) and immunofluorescence staining (representative staining in Figure 3E–3G), suggesting reduced *TRPM7* could alleviate malignancy of the BCa cells.

### Induction of ROS in BCa cells with *TRPM7* deficiency

The status of ROS in the BCa cells (T24 and EJ) were measured by flow cytometry analysis (Supplementary Figure S2A) and fluorescence staining (Supplementary Figure S2C). Statistical analysis (Supplementary Figure S2B) revealed a significantly increase ROS in the *siTRPM7*-treated BCa cells by the flow cytometry measurement. Western blot analysis indicated an increased protein abundance of Catalase and SOD2 in the BCa cells with downregulated *TRPM7* (Supplementary Figure S2D).

### *TRPM7* deficiency impaired BCa cells motility and invasion via the PI3K/AKT pathway

**Figure 4:**
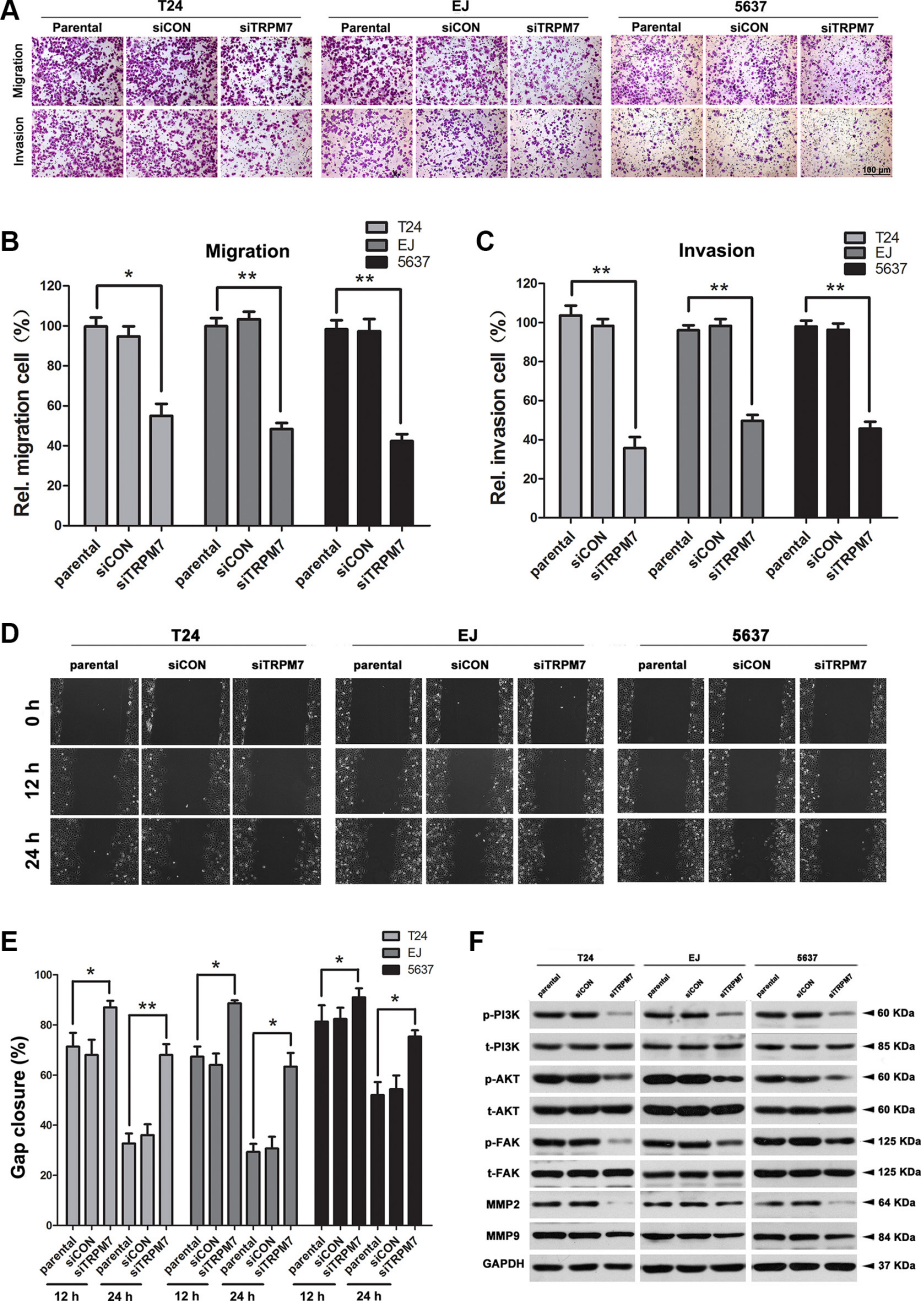
*TRPM7* deficiency inhibited BCa cells migration and invasion through PI3K/AKT pathway (**A**) Cell migration and invasion were evaluated by transwell assay for the parental, *siCON-* and *siTRPM7*-treated BCa cells by the transwell chamber system. Migrative and invasive cells were fixed and stained with crystal violet. Scale bar is 100 μm. The relative cell number of migration (**B**) and invasion (**C**) in each cells was statistically analyzed. Cell types and treatment of *siRNA* were indicated. (**D**) Migration of parental, *siCON-* and *siTRPM7*-treated BCa cells was determined at 0, 12 and 24 h by wound healing assay and the gap closure was statistically analyzed (**E**). All values for statistical analysis shown were mean ± SD of triplicate measurements and repeated three times with similar results. Statistical significance was calculated using ANOVA. *p < 0.05, **p < 0.01. (**F**) Western blot analysis for protein abundance of phosphorylated and total PI3K, AKT, FAK, as well as MMP2/9 in parental, *siCON*- and *siTRPM7-*treated BCa cells. GAPDH was used as a loading control (cell types, treatment of *siRNA* and protein masses were indicated).

The relationship between TRPM7 and BCa cell motility and invasion was investigated using transwell and wound healing assays. Transwell assay suggested that knockdown of *TRPM7* in BCa cells could reduce cell migration and invasion (Figure 4A), which was confirmed by statistically analysis in Figure 4B–4C. Moreover, wound healing assay revealed that *TRPM7* deficiency in BCa cells could reduce the number of migrated cells (Figure 4D). The gap closure (%) was statistically analyzed (Figure 4E). Furthermore, downregulation of p-FAK and MMP2/9 in BCa cells with *siTRPM7* was noticed (Figure 4F). Interestingly, we have also observed the PI3K/AKT signaling pathway, a downstream of FAK [42], was affected by *TRPM7* deficiency as well, revealing a strong reduction of p-PI3K and p-AKT (Figure 4F).

### Knockdown of *TRPM7* triggered G0/G1 phase cell cycle arrest and apoptosis in the BCa cells

**Figure 5:**
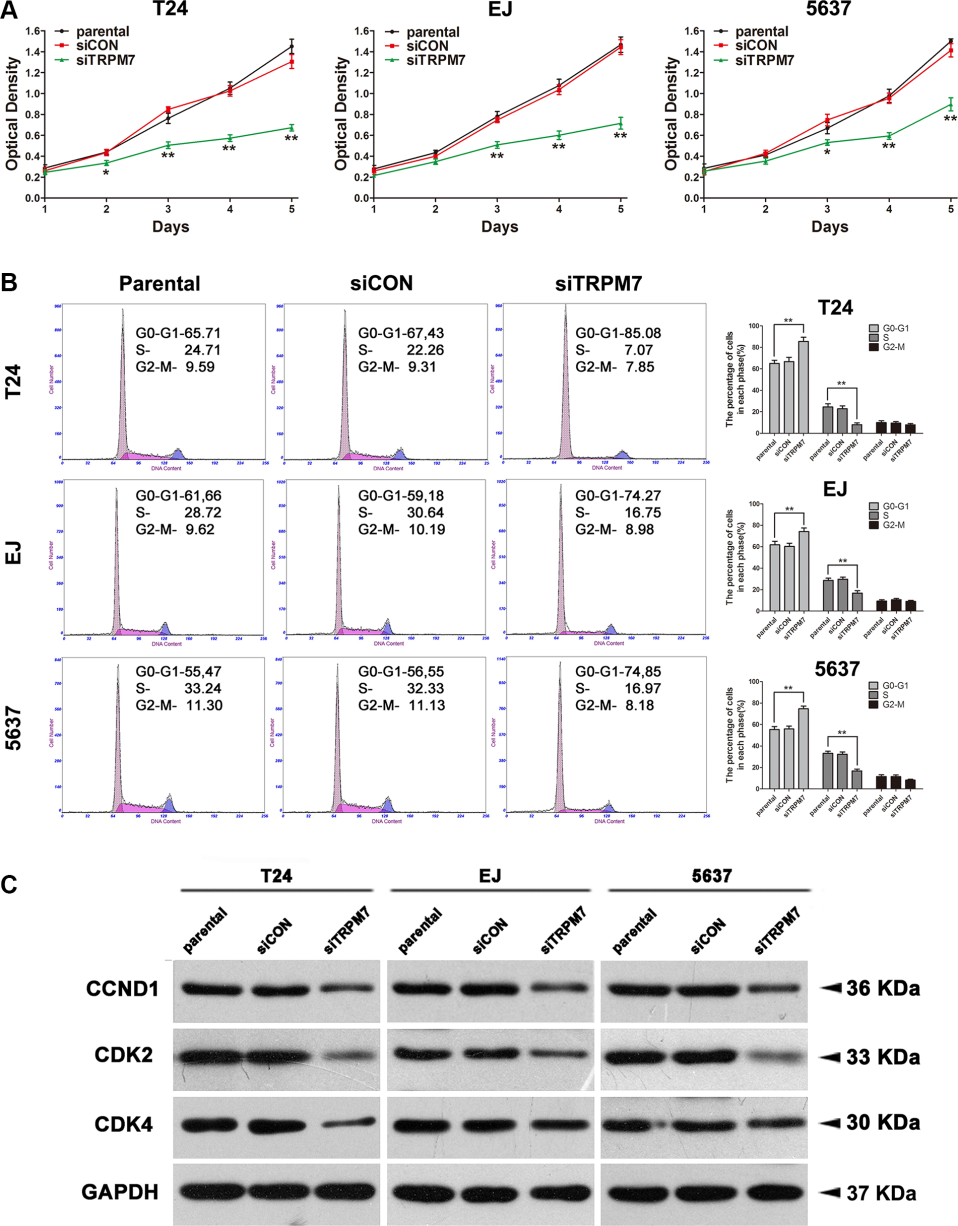
Downregulation of *TRPM7* repressed cell proliferation by triggering cell cycle arrest at G0/G1 phase in the BCa cells (**A**) Viability of cells transfected with *siTRPM7* from day 1 until day 5 was analyzed by CCK-8 assay, comparing with *siCON* and parental cells. (**B**) Flow cytometry analysis for the BCa cells treated with *siTRPM7* for 48 h compared with *siCON* and parental cells. Percentages (%) of cell populations at different stages of cell cycles were listed within the panels. All histograms revealed the percentage (%) of cell populations from three independent experiments. Cell types and treatment of *siRNA* were indicated. *p < 0.05, **p < 0.01. (**C**) Downregulation of protein abundance involved in the cell cyle regulation (CDK2, CDK4 and CCND1, also written as Cyclin D1) in the BCa lacking *TRPM7* was revealed by Western blot analysis. GAPDH abundance was used as a control. Cell types, treatment of *siRNA* and protein masses were indicated.

**Figure 6:**
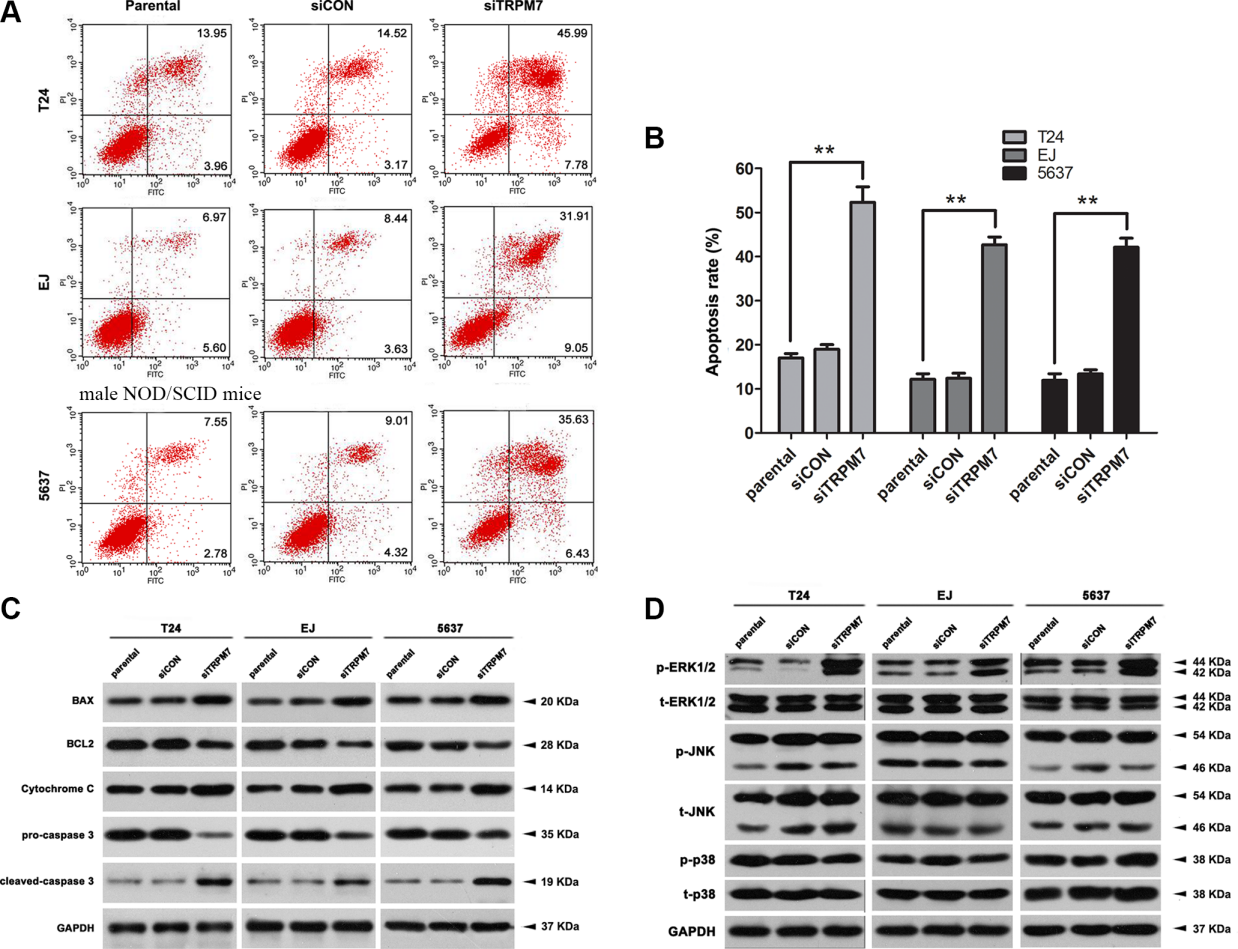
*TRPM7* deficiency induced BCa cell apoptosis via ERK1/2 pathway (**A**) Representative flow cytometry analysis of apoptosis stained with Annexin V and PI in parental, *siCON-* and *siTRPM7*-transfected BCa cells. (**B**) Statistical analysis of apoptotic rate (%) in the three BCa cell lines after the *siRNA* treatment. Cell types and treatment of *siRNA* were indicated. The values shown were mean ± SD of triplicate measurements and repeated three times with similar results. Statistical significance was calculated using ANOVA. **p <0.01. **(C)** Western blot analysis for proteins involvement in mitochondrial dependent apoptotic pathway, such as BAX, BCL2, Cytochrome C and related proteins pro-caspase 3 and cleaved-caspase 3. **(D)** Proteins in the MAPK family were affected indicated by Western blot analysis. Phosphorylated and total ERK1/2, JNK and p38 at protein level was analyzed, suggesting a dominated activation of ERK1/2 in the MAPK family by the *siTRPM7*. GAPDH abundance was used as a control. Cell types, treatment of *siRNA* and protein masses were indicated.

CCK-8 assay revealed that *TRPM7* deficiency induced inhibition of cell growth, compared with the parental and *siCON* cells (Figure 5A). To better understand the underlying mechanism, the effects of *TRPM7* on cell cycle (Figure 5B) and apoptosis (Figure 6A–6B) were analyzed using flow cytometry analysis. Compared with the control cells, knockdown of *TRPM7* in the BCa cells triggered cell cycle arrest at G0/G1 phase (statistically analyzed in Figure 5B) as well as a reduction in the proportion of cells in S phase. Indeed, cell cycle related proteins such as Cyclin D1 and CDK2/4 were decreased in the BCa cells with *TRPM7* knockdown (Figure 5C). Moreover, *TRPM7* deficiency resulted in a significant increase of the apoptotic BCa cells (Figure 6A–6B). We have analyzed the alterations of proteins involved in the apoptosis by Western blot (Figure 6C–6D), exhibiting upregulation of the apoptosis inducer BAX [43, 44] and downregulation of the apoptosis inhibitor BCL2 [45, 46] in the *siTRPM7*-treated BCa cells. Furthermore, pro-caspase 3, a downstream protein of BCL2 and BAX in the apoptotic cascade [47], was decreased, and its active form cleaved-caspase 3 was increased (Figure 6C). Moreover, we have found that Cytochrome C was also upregulated, suggesting that TRPM7 deficiency could trigger BCa cell apoptosis through a mitochondrial-dependent manner [48] (Figure 6C). Another key family, the MAPK including ERK1/2, JNK and p38, which are involved in the development and apoptosis regulation of tumor cells, was also altered in the *siTRPM7*-treated BCa cells (Figure 6D). TRPM7 deficiency strongly induced phosphorylated ERK1/2 (p-ERK1/2) in the BCa cells, with only a mild impact on p-JNK and p-p38.

### Downregulation of *TRPM7* induced BCa cell apoptosis through activation of ERK1/2

**Figure 7:**
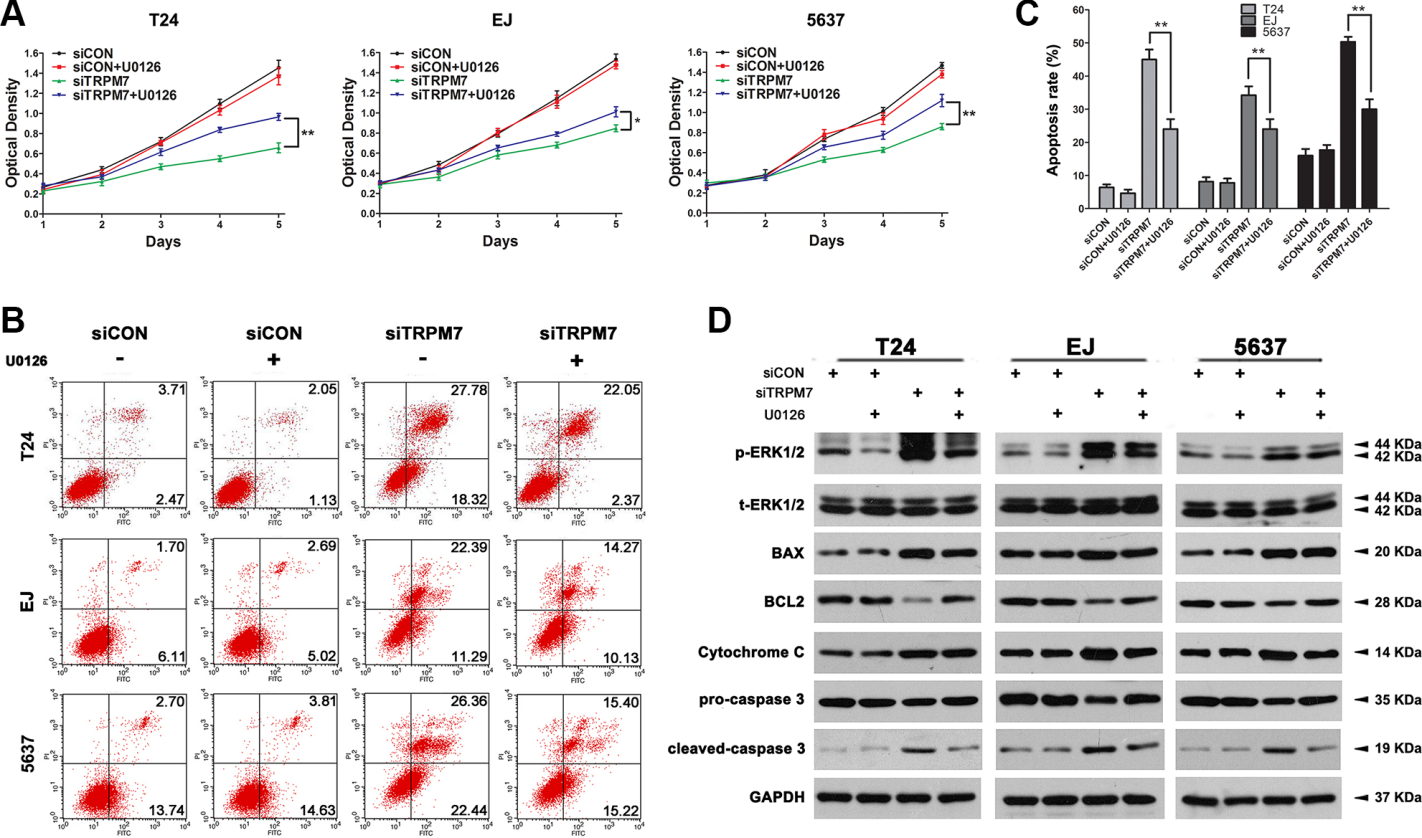
Recovering *siTRPM7*-induced BCa cell apoptosis by U0126 to deactivate ERK1/2 (**A**) T24, EJ, 5637 cells were pre-treated by U0126 at 10 mM for 2 h and treated by *siTRPM7* for 48 h, comparing with *siCON* and parental cells. Proliferation of the BCa cells were analyzed by CCK-8 assay. Cell types and treatment of *siRNA* were indicated. *p < 0.05, **p < 0.01. (**B**) Apoptotic cells staining with Annexin V and PI were revealed by flow cytometry analysis and the apoptotic rates were statistically analyzed (**C**). Values of statistical data shown were mean ± SD of triplicate measurements and repeated three times with similar results. Statistical significance was calculated using ANOVA. *p < 0.05, **p < 0.01. (**D**) Western blot analysis for phosphorylated and total ERK1/2 as well as mitochondrial dependent apoptotic pathway proteins (BAX, BCL2, Cytochrome C, pro-caspase 3 and cleaved-caspase 3). GAPDH abundance was used as a control. Cell types, treatment of *siRNA* and protein masses were indicated.

We have pre-treated the BCa cells using U0126 to repress the activity of ERK1/2 and transfected with *siTRPM7* and controls, revealing a significant recovery of cell growth delay triggered by *TRPM7* deficiency in all the three BCa cells indicated by the CCK-8 assay (Figure 7A). Importantly, flow cytometry analysis exhibited that deactivation of ERK1/2 through pre-treatment with the U0126 (Figure 7B), could reverse *TRPM7* deficiency induced apoptosis (statistically analyzed in Figure 7C), as demonstrated by reduced p-ERK1/2 and reversed BAX/BCL2 ratio, as well as decreased Cytochrome C and cleaved-caspase 3 using Western blot analysis (Figure 7D).

### Reduction of either TRPM7 protein activity by carvacrol or *TRPM7* gene transcription by *lentiviral-shRNA* could inhibit bladder cancer growth *in vivo*

**Figure 8:**
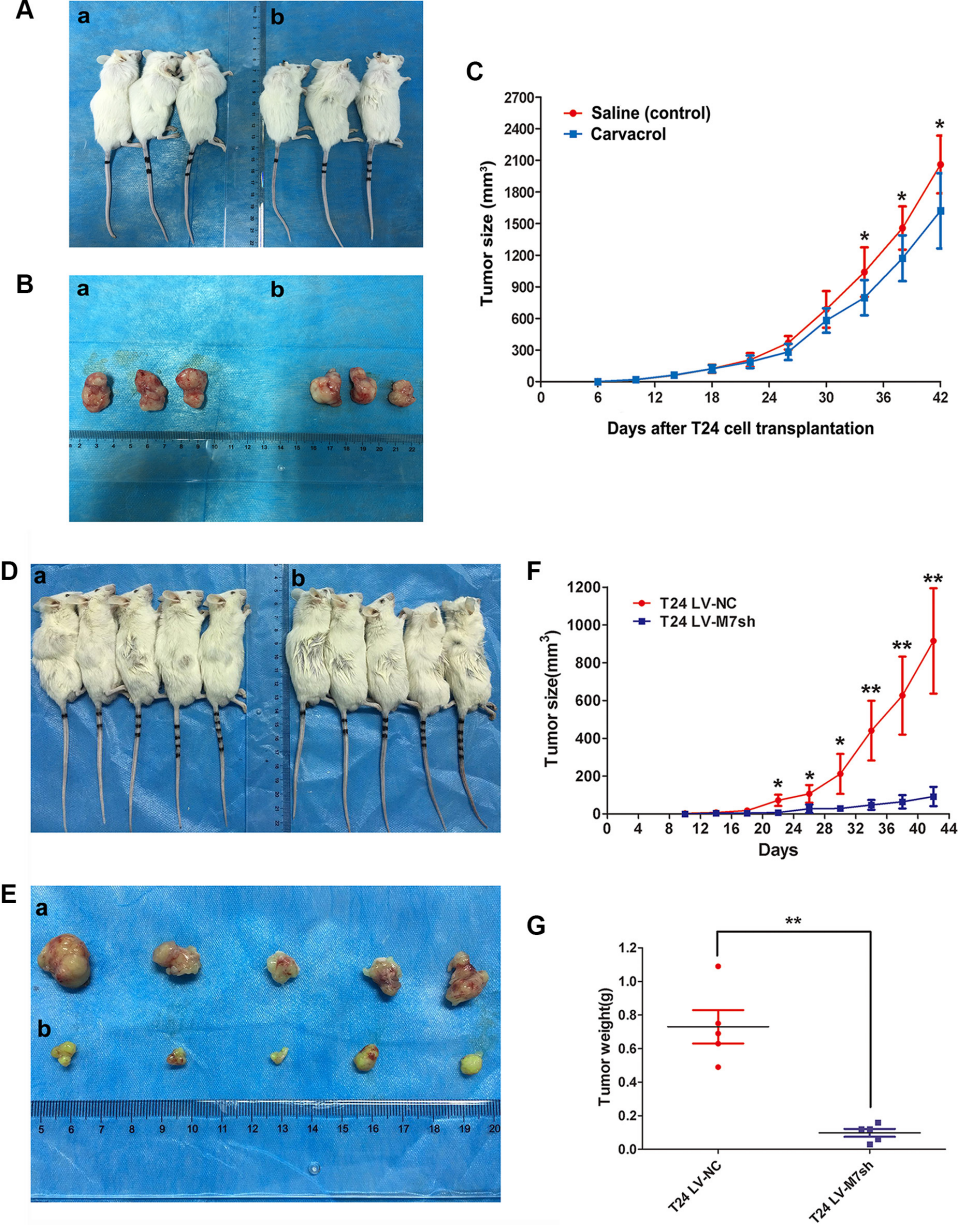
Deactivation of TRPM7 by carvacrol and decreased *TRPM7* by *lentiviral-shRNA* suppressed bladder cancer growth *in vivo* (**A**) Nude mice were subcutaneously transplanted with T24 cell for 14 days, and continueouslly intraperitoneally injected by carvacrol for 28 days (b) compared with injection by saline (a) as a control. (**B**) Dissected tumor from the nude mice injected by carvacrol (b) and saline (a). (**C**) Statistical analysis of tumor size (mm^3^) measured by a caliper and calculated using *t-test*, *p < 0.05. Days after T24 cell transplantation, tumor size and saline / carvacrol injection were indicated. (**D**) Nude mice were subcutaneously transplanted with T24 cell infected by *lentiviral-TRPM7-shRNA* (*T24 LV-M7sh*) for 42 days (b) compared with *lentiviral-control-shRNA* (*T24 LV-NC*) (a). (**E**) Dissected tumor from the nude mice injected by *lentiviral-TRPM7-shRNA* (*T24 LV-M7sh*) (b) and *lentiviral-control-shRNA* (*T24 LV-NC*) (a). Statistical analysis of tumor size (**F**) and tumor weight (**G**), calculated using *t-test*, *p < 0.05, **p < 0.01. Days after T24 cell transplantation, tumor size and types of *lentiviral-shRNA* infection were indicated.

To further analyze the effects of TRPM7 activity in BCa tumorigenesis *in vivo*, a NOD/SCID mouse model transplanted with T24 cells was established (Figure 8A– 8B). Two weeks after the transplantation, carvarcrol was intraperitoneally injected in the mice to inhibit the activity of TRPM7 (Figure 8A–8B, b). In contrast, other mice transplanted with T24 were injected by saline as control (Figure 8A–8B, a). The difference of tumor size for the carvacrol treated mice and the control mice was statistically analyzed in Figure 8C, suggesting a reduced tumor growth by the deactivation of TRPM7 protein *in vivo*. In addition, we also transplanted the T24 cells infected with *lentiviral-TRPM7-shRNA* (*T24 LV-M7sh*) to observe the tumor growth under downregulation of *TRPM7* at transcriptional level (Figure 8D–8E). We noticed significantly decreased tumor size (Figure 8F) and weight (Figure 8G) triggered by *TRPM7* deficiency comparing with the control group (*T24 LV-NC*).

### Overexpression of *TRPM7* induced BCa cell proliferation and motility

BCa cells (T24 and EJ) were transfected with pcDNA5/FRT/TO/HA-TRPM7 vector to overexpress *TRPM7* (Supplementary Figure S3A), which could trigger significantly increased cell proliferation revealed by CCK- 8 assay (Supplementary Figure S3B) and clonogenic survival assay (Supplementary Figure S3C). Induction of T24 and EJ cell migration rates was suggested by transwell migration assay, confirmed by the statistically analysis (Supplementary Figure S3D).

## DISCUSSION

The TRPM family, an essential family of proteins exhibited distinct functions on tumorigenesis [49, 50]. Our group has reported that the TRPM family could regulate cell cycle and growth in human osteosarcoma and prostate cancer cells [32, 51]. The present study aims to investigate the effect and mechanism of TRPM7, an important TRPM family member involved in Ca^2+^-permeable channel, on human bladder cancer, which remains largely unknown yet. Microarray analysis using human bladder cancer tissues compared with normal bladder epithelium tissues has suggested a close correlation between calcium signaling pathway and bladder cancer via MAPK signaling pathway, confirmed by overrepresentation using microarray raw data and DAVID database. We analyzed gene expression and protein abundance of TRPM7 in BCa tissues, observing a strong upregulation of *TRPM7* at the gene expression and protein level by double immunofluorescence staining analysis, co-localized with OCT-4, a potential marker for BCa cells [52, 53]. Moreover, we noticed the upregulation of *TRPM7* may be correlated with the EMT markers in the BCa tissue, which could reveal the malignancy of tumor [54, 55].

We have selected three typical BCa cell lines with distinguished malignancy, T24, EJ and 5637, to generate the BCa cell model with *TRPM7* deficiency using *siRNA* transfection. Indeed, we confirmed that knockdown of *TRPM7* in the three BCa cell lines at gene expression and protein levels. Moreover, downregulation of *TRPM7* resulted in a reversed status for the EMT markers, suggesting deficiency of *TRPM7* could alleviate malignancy of BCa. Furthermore, our results revealed a significant increase of ROS status and protein abundance involved in the ROS metabolism in the BCa cells with downregulated *TRPM7*, suggesting a correlation between TRPM7 and ROS in BCa cells.

Knockdown of *TRPM7* could reverse EMT markers in the three BCa cells, and we also observed that *TRPM7* deficiency could also inhibit migration and invasion of BCa cells, as EMT has been reported to play a critical role in cancer cell migration and invasion [56]. To confirm the effect triggered by *siTRPM7* treatment, we have established a BCa cell model with *TRPM7*-overexpression and noticed the migration rate was significantly increased. Moreover, treatment of *siTRPM7* could also reduce MMP2/9 and phosphorylated FAK, PI3K, AKT in the BCa cells. Recent studies suggested FAK is a participant in focal adhesion dynamics between cells, playing a key role in cell motility [57] and MMPs could degrade the extracellular matrix and promote cancer cells to metastasize [58, 59]. Another publications reported that PI3K/AKT is a key signaling pathway [60, 61], which was deactivated by the *TRPM7* deficiency in our study as well, suggesting that inhibited BCa cell motility triggered by *TRPM7* knockdown could be via the PI3K/AKT signaling pathway.

A major effect of TRPM7 was to induce delayed cell proliferation, cycle arrest at G0/G1 phase and apoptosis in the BCa cells with *TRPM7* deficiency. Proteins involved in cell cycle regulation, CCND1 and CDK2/4, were all reduced after the *siTRPM7* treatment. Moreover, proteins involved in apoptosis were strongly altered, especially imbalance of the BAX/BCL2 ratio, which is involved in mitochondrial-dependent apoptosis [62, 63], and related proteins including cleaved-caspase 3 [64, 65] and Cytochrome C [66, 67], suggesting that *TRPM7* deficiency could trigger a mitochondrial-mediated apoptosis. Importantly, as suggested by our microarray analysis, MAPK signaling pathway, a central regulator in the pathway network, could be altered by the affected calcium signaling pathway due to *TRPM7* knockdown. Western blot analysis indicated ERK1/2, a member of MAPK family, was remarkably increased in the phosphorylated form, suggesting knockdown of *TRPM7* might induce a mitochondrial dependent apoptosis via ERK1/2, which could be activated by phosphorylation on specific sites and promote either intrinsic or extrinsic apoptotic pathways by induction of mitochondrial Cytochrome C release or caspase-8 activation, permanent cell cycle arrest or autophagic vacuolization [68].

Therefore, we deactivated ERK1/2 by pre-treatment of U0126 in the three BCa cells and noticed a decrease of the p-ERK1/2 at protein level. Furthermore, the delayed cell growth caused by *siTRPM7* was reversed significantly, and the apoptotic cell rates were significantly reduced by ERK1/2 inhibition, as well as the proteins involved in the apoptosis pathway were recovered to the level of the control group. Importantly, we have established a T24-transplanted NOD/SCID mouse model with carvacrol [69] and *lentiviral-shRNA* treatment respectively, observing a significant delayed tumor growth by both reduction of TRPM7 protein activity and *TRPM7* gene transcription. Therefore, our results *in vitro* and *in vivo* have revealed that the downregulation of *TRPM7* could reduce the BCa cell proliferation, trigger cell cycle arrest and apoptosis. In conclusion, downregulated *TRPM7* could reduce the activity of bladder cancer cells and induce cell apoptosis via ERK1/2 pathway.

## MATERIALS AND METHODS

### Ethical statement for human bladder tissue samples

Three stage II bladder cancer tissue samples and three normal bladder tissue samples were collected from male patients (ages 62 ± 1.581) after surgery by radical resection and male donors (ages 37 ± 2.327) undergoing transplant by accidental death, at Zhongnan Hospital of Wuhan University, Wuhan, China. Informed consent was obtained from all subjects to collect the samples from the operating room and to store them in liquid nitrogen for total RNA isolation, as well as to fix the samples in 4% Paraformaldehyde (PFA) for immunofluorescence staining analysis. The study was approved by the Ethics Committee at Zhongnan Hospital of Wuhan University (approval number: 2015029, Supplementary Information S1) and the sample collection as well as treatment were carried out in accordance with the approved guidelines.

### Ethical statement for NOD/SCID mice

Investigation has been conducted in accordance with the ethical standards and according to the Declaration of Helsinki and according to national and international guidelines and has been approved by the author's institutional review board. Male NOD/SCID mice in this study were purchased from Beijing HFK Bioscience Co., Ltd. in Beijing, China (http://www.hfkbio.com/en/).

### Human bladder cancer cell lines

Human bladder cancer cell lines EJ cells (carcinoma, Cat. #CL-0274) and BIU-87 (Cat. #CL-0035) were purchased from the Procell Co., Ltd. in Wuhan, China. The T24 (transitional cell carcinoma, Cat. #SCSP-536), 5637 (grade II carcinoma, Cat. #TCHu1), UM-UC-3 (Cat. #TCHu217), RT-4 (Cat. #TCHu226) and SV-HUC-1 (Cat. #TCHu169) were kindly provided by the Stem Cell Bank, Chinese Academy of Sciences in Shanghai, China. Identification of the BCa cell lines was at the China Centre for Type Culture Collection in Wuhan, China. The EJ, BIU-87, T24, 5637 and SV-HUC-1 cells were cultured in RPMI-1640 medium (Gibco, China), UM-UC-3 cells were cultured in DMEM medium (Gibco, China) and RT-4 cells were cultured in McCoy's 5A Medium (Gibco, China) containing 1% penicillin G sodium/streptomycin sulphate and 10% fetal bovine serum (FBS) (Gibco, Australia) in a humidified atmosphere consisting of 95% air and 5% CO_2_ at 37°C.

### RNA expression analyses

### Total RNA isolation from bladder tissues and BCa cells

Total RNA was isolated from bladder tissues and distinct BCa cells using the Qiagen RNeasy Mini Kit (Cat #74101), combined with QIAshredder from Qiagen (Cat #79654) using a centrifuge (Eppendorf, Cat #5424) to increase the quantity and quality of isolated total RNA, according to the manufacturer's protocol. Each RNA sample was digested by DNase I (RNase-Free DNase Set, Qiagen, Cat #79254) to remove possible contamination of genomic DNA. The quantity of isolated RNA was measured by a NanoDrop^®^ ND-1000 UV-Vis spectrophotometer (Thermo Scientific, USA).

### Microarray analysis of mRNA isolated from human bladder tissues

Our group has established a microarray analysis using mRNA isolated from the three stage II bladder cancer tissues and the three normal bladder tissues, as described by Wang *et al.* in 2016 [2]. Briefly, according to the standard Affymetrix protocol, 250 ng total RNA of each sample was prepared to biotinylated cDNA by Ambion^®^ WT Expression Kit. On GeneChip Human Transcriptome Array 2.0, 5.5 μg of cDNA were hybridized for 16 h at 45°C, continuously washed and stained in the Affymetrix Fluidics Station 450, scanned by using Affymetrix^®^ GeneChip Command Console (AGCC) installed in GeneChip^®^ Scanner 3000 (7G). Data analysis was with a Robust Multichip Analysis (RMA) algorithm using Affymetrix default analysis settings and global scaling as normalization method. By Gene ontology (GO) and pathway-Relation-Network (Path-net) analysis tools based on Kyoto Encyclopedia of Genes and Genomes (KEGG) Pathway Database using Gene Cloud of Biotechnology Information (GCBI Plateform, Shanghai, China) [70], genes and pathways correlated with bladder cancer were generated. The resulting gene list was subjected to the Database for Annotation, Visualization and Integrated Discovery (DAVID) [71] as well for annotation and overrepresentation analysis of the genes involved in calcium signaling pathway (Supplementary Figure S2). The microarray data was submitted to the Gene Expression Omnibus (GEO) database with accession number GSE76211. All data are MIAME compliant.

### Reverse transcription and quantitative real time PCR (qRT-PCR)

1 μg of total RNA isolated from bladder tissues or BCa cells was mixed with oligo (dT) 12–18 primers to synthesize first-strand cDNA by using RevertAid First Strand cDNA Synthesis Kit (Thermo Scientific, China). 1 μg cDNA were used for each reaction of the polymerase chain reactions (PCR) in a final volume of 20 μl. All primers conducted with the SYBR Premix Ex Taq II (Takara Bio, China) were tested for optimal annealing temperatures and PCR conditions were optimized with gradient PCRs on a Bio-Rad iCycler (Cat. #CFX96). Primer sequences and annealing temperatures are summarized in Table 1. Values were normalized for amplified β-actin alleles. Relative gene abundance = 2^−ΔΔct^, Δct = ct_target gene_ - ct_β-actin_, for BCa cells ΔΔct = Δct_siRNA-treated_ - Δct_siRNA-untreated_, for bladder tissues ΔΔct = Δct_BCa tissues_ - Δct_normal bladder tissues_ (ct = threshold cycle).

**Table 1:** List of primers for qRT-PCR

Gene	Symbol	Forward primer (5′–3′)	Reverse primer (5′–3′)	Annealing temperature (°C)	Length (bp)
Transient receptor potential melastatin 7	*TRPM7*	5′- TGGATGATGGC ACTGTTGGAA −3′	5′- CATTTGGCCC ACCCTCAAATATAA −3′	56	144
Actin, beta	*ACTB*	5′- AGAGCTACGA GCTGCCTGAC −3′	5′- AGCACTGTGTT GGCGTACAG −3′	56	184
calcium/calmodulin dependent protein kinase II beta	*CAMK II B*	5′- GCACACCA GGCTACCTGTC −3′	5′- GGACGGGAAG TCATAGGCA −3′	56	179
calmodulin 1	*CALM1*	5′- TTGACTTCCCC GAATTTTTGACT −3′	5′- GGAATGCCTCA CGGATTTCTT −3′	56	81
calmodulin 3	*CALM3*	5′- GACCATTGAC TTCCCGGAGTT −3′	5′- GATGTAGCCATT CCCATCCTTG −3′	56	118
protein phosphatase 3 catalytic subunit alpha	*PPP3CA*	5′- CCAAGTCACC GGCTTACAG −3′	5′- CCTCCTTCATAA GATGCGCCTT −3′	56	88
protein phosphatase 3 catalytic subunit beta	*PPP3CB*	5′- CCCCAACACATC GCTTGACAT-3′	5′- GGCAGCACCCT CATTGATAATTC −3′	56	140
protein phosphatase 3 catalytic subunit gamma	*PPP3CC*	5′- ACCGCGTCATC AAAGCTGT-3′	5′- CTTCCAGTCGT CCTTCCTTTAC −3′	56	125

### Cell culture experiments

### Knockdown and overexpression of *TRPM7* in the BCa cells

*TRPM7*-target specific small interfering RNA (*siRNA*) and lentiviral small hairpin RNA (*LV-shRNA*) were synthesized by Genepharma Ltd. in Suzhou, China. Distinct BCa cells (T24, EJ and 5637) were transfected with *TRPM7-siRNA* (*siTRPM7*) using lipofectamine 2000 (Invitrogen, USA), according to the manufacturer's protocol. The sense sequence of *TRPM7-siRNA* (*siTRPM7*) */ TRPM7-shRNA* (*shTRPM7*) was 5′-GUCUUGCCAUGAAAUACUCTT-3′, and the sense sequence of *control-siRNA* (*siCON*) */ control-shRNA* (*shCON*) was 5′-UUCUCCGAACGUGUCAGGUTT-3′. After transfection by *siTRPM7* for 48 h, alterations of *TRPM7* at transcriptional and protein levels were evaluated by the qRT-PCR, Western blot and immunofluorescence staining analysis. The BCa cells infected by *lentiviral*-*TRPM7*-*shRNA* (*LV-M7sh*) and *lentiviral-control-shRNA* (*LV-NC*) were treated by 0.8 mg/ml puromycin (Sigma, USA) for 14 days to select the antibiotic-resistant cells xenotransplanted into NOD/SCID mice.

The plasmid of *TRPM7*-overexpression (pcDNA5/FRT/TO/HA-TRPM7 vector) was a gift from Professor Loren W. Runnels at University of Medicine & Dentistry of New Jersey, USA. The cell model of *TRPM7*-overexpression was established according to Su *et al.* [72] from the group of Professor Runnels. Briefly, the BCa cells (EJ and T24) were transfected by the pcDNA5/FRT/TO/HA-TRPM7 vector and induced by 1 mg/ml tetracyclines for 48 h.

### Pre-treatment using MKK inhibitor for rescue experiments

Before *siRNA* transfection, BCa cells were pre-treated by MKK inhibitor U0126 (Sigma, USA) at 10 mM for 2 h to deactivate ERK1/2 [13, 73, 74]. BCa cells in the untreated group were pre-incubated with appropriate amount of vehicle (0.1% DMSO). Both groups were submitted for the RNA interference and alterations of cell viability and apoptosis were measured by CCK-8 assay and flow cytometry analysis, respectively.

### Wound healing assay

When the BCa cells grew to 95% confluence, a 200 μl pipette tip was used to scratch a wound to cell monolayer. After scratching, cells were washed with PBS several times to remove non-adherent debris, then 0.5% FBS medium was added to allow cells to move into the gap without the influence of serum. Four different equidistant points of the scratched area were photographically measured and imaged by an inverted phase contrast microscope (Leica, Cat. #DMI 1) at 0 h, 12 h and 24 h. Migration rate was calculated as the proportion of initial scratch distant of each sample using the mean distance between both borderlines that remain cell-free after cell migration.

### Transwell migration assay

A 24-well plate transwell chamber system (Corning, USA) with 8.0 μm pore size was used. Cells were suspended in 0.5% FBS medium at a density of 5 × 10^5^ cell/ml and 100 μl cell suspension was seeded in the upper chamber insert without ligand, while the lower chamber was filled with 10% FBS medium. After 24 h incubation at 37°C, cells on the upper insert were removed by cotton swabs, and cells that migrated to the lower side were fixed with 4% PFA and stained with crystal violet. Then the chambers were placed under an inverted phase contrast microscope and 16 random areas were selected to observe and count the migrated cells.

### Transwell invasion assay

In transwell invasion analysis, 40 μl ECM gel solution (Sigma, USA) diluted to a concentration of 1 mg/ml was coated on the surface of the upper insert of Transwell chamber and placed in a 37°C incubator for overnight. BCa cells were then suspended in 0.5°C FBS medium at a density of 8 × 10^5^ cell/ml and 100 μl cell suspension was seeded in the upper chamber insert. After incubation 48 h at 37°C, cells were fixed, stained and counted under an inverted phase contrast microscope.

### CCK-8 assay

Cell Counting Kit-8 (Roche Biochemicals, Germany) was used for cell viability measurement. 100 μl cell suspensions (2 × 10^4^ cells per ml) with 10% FBS medium was seeded to a 96-well plate and incubated for 5 days at 37°C. Then, 10 μl CCK-8 reagent was added to each well at the indicated time and incubated for 4 h at 37°C. Absorbance was measured at 450 nm by a Rayto-6000 system (Rayto, China).

### Clonogenic survival assay

BCa cells were seeded in 6-well plates (1500 cells per well) and grew into colonies for approximately 15 days. Colonies were emerged and fixed by 4% PFA for 30 min, staining with crystal violet, counted and defined as aggregates of 50 or more cells and photographed.

### Flow cytometry analysis for alterations of cell cycle and apoptosis

For cell cycle analysis, 1 × 10^6^ cells were harvested and fixed in 70% ice cold ethanol at −20°C for overnight. After centrifugation, pellets were resuspended with PBS containing 50 μg/ml propidium iodide (Sigma-Aldrich, USA) and 0.1 mg/ml RNaseA (20 μg/ml in PBS) in the dark. After incubation at 37°C for 30 min, the DNA content distribution was analyzed by flow cytometry analysis (Beckman, Cat. #FC500). For apoptosis analysis, after transfection for 48 h, cells were collected, washed with PBS, and stained with FITC Annexin V Apoptosis Detection Kit I (BD biosciences, USA) and analyzed by the flow cytometry analysis.

### ROS detection by staining with DCFH-DA

The fluorescent probe 2′,7′-Dichlorofluorescin diacetate (DCFH-DA) was used to evaluate intracellular ROS levels. BCa cells transfected with *siTRPM7* and *siCON* were used for this experiment. After transfection of cells and growth for 48 h, 10 μmol of DCFH-DA (Sigma, USA) was added to 2 ml medium, and incubated at 37°C for 30 min. Thereafter, the cells were washed three times with PBS and submitted to flow cytometry analysis. For ROS staining, slides with the BCa cells were stained by DCFH-DA and incubated for 30 min at room temperature, then washed by PBS three times. Nuclei were counterstained with 1 μM DAPI for 20 min at room temperature. Images were taken with a fluorescence microscope (Olympus, Cat. #IX73).

### Protein analyses

### Isolation of total protein from BCa cells and Western blot analysis

The BCa cells (T24, EJ and 5637) were sonicated and lysed in RIPA buffer containing protease inhibitor and phosphatase inhibitor (Sigma-Aldrich, USA) on ice for 30 min, then centrifuged at 12,000 g for 15 min to collect supernatant. By Bradford protein assay (Bio-Rad, Germany) the concentrations of protein were determined using Bovine serum albumin (BSA) as standard. The isolated total protein was resolved using 6–15% SDS-PAGE and transferred to PVDF membrane (Millipore, USA). Membranes were blocked by 5% non-fat milk and incubated with primary antibodies (Table 2) at 4°C for overnight. After washing, the membranes were incubated with secondary antibody (listed in Table 3) at room temperature for 2 h. Bands were visualized using an enhanced chemiluminescence (ECL) kit (Bio-rad, USA) and detected by Kodak Biomax MR films.

**Table 2:** List of primary antibodies

Antigens	Species antibodies raised in	Dilution (IF)	Dilution (WB)	Supplier
Akt (pan), mouse	Rabbit, monoclonal	-	1:2,000	Cell Signaling Technology, USA, Cat. #4691
Bax, human	Rabbit, monoclonal	-	1:2,000	Cell Signaling Technology, USA, Cat. #5023
Bcl-2, human	Rabbit, monoclonal	-	1:2,000	Cell Signaling Technology, USA, Cat. #2872
Caspase-3, human	Rabbit, monoclonal	-	1:2,000	Cell Signaling Technology, USA, Cat. #9665
Cytochrome c, human	Rabbit, monoclonal	-	1:500	Cell Signaling Technology, USA, Cat. #4280
CDK2, human	Rabbit, monoclonal	-	1:2,000	Cell Signaling Technology, USA, Cat. #2546
CDK4, human	Rabbit, monoclonal	-	1:2,000	Cell Signaling Technology, USA, Cat. #12790
Cleaved Caspase-3, human	Rabbit, monoclonal	-	1:500	Cell Signaling Technology, USA, Cat. #9664
Cyclin D1, human	Rabbit, monoclonal	-	1:2,000	Cell Signaling Technology, USA, Cat. #2978
E-cadherin, human	Rabbit, monoclonal	1:200	1:500	Cell Signaling Technology, USA, Cat. #3195
FAK, human	Rabbit, monoclonal	-	1:1,000	Cell Signaling Technology, USA, Cat. #13009
Glyceraldehyde 3-phosphate dehydrogenase (GAPDH), human	Mouse, monoclonal	1:200	1:2,000	Santa Cruz Biotechnology Inc., USA, Cat. #sc-365062
MMP-2, human	Rabbit, monoclonal	-	1:500	Cell Signaling Technology, USA, Cat. #13132
MMP-9, human	Rabbit, monoclonal	-	1:1,000	Cell Signaling Technology, USA, Cat. #13667
N-cadherin, human	Rabbit, monoclonal	1:200	1:1,000	Cell Signaling Technology, USA, Cat. #13116
OCT-4, human	Rabbit, monoclonal	1:200	1:1,000	Cell Signaling Technology, USA, Cat. #2750
OCT-4, human	Mouse, monoclonal	1:200	-	Novus Biologicals, USA, Cat. #NB110-90606
p38 MAPK, human	Rabbit, monoclonal	-	1:2,000	Cell Signaling Technology, USA, Cat. #8690
p44/42 MAPK (Erk1/2), rat	Rabbit, monoclonal	-	1:2,000	Cell Signaling Technology, USA, Cat. #4695
Phospho-Akt (Ser473), human	Rabbit, monoclonal	-	1:1,000	Cell Signaling Technology, USA, Cat. #4060
Phospho-FAK (Tyr397), human	Rabbit, monoclonal	-	1:1,000	Cell Signaling Technology, USA, Cat. #8556
Phospho-p38 (Thr180/Tyr182), human	Rabbit, monoclonal	-	1:1,000	Cell Signaling Technology, USA, Cat. #4511
Phospho-p44/42 MAPK (Erk1/2) (Thr202/Tyr204), human	Rabbit, monoclonal	-	1:1,000	Cell Signaling Technology, USA, Cat. #4370
Phospho-PI3 Kinase p85 (Tyr458)/p55 (Tyr199), mouse	Rabbit, monoclonal	-	1:1,000	Cell Signaling Technology, USA, Cat. #4228
Phospho-SAPK/JNK (Thr183/Tyr185), human	Rabbit, monoclonal	-	1:1,000	Cell Signaling Technology, USA, Cat. #4668
PI3 Kinase p85, human	Rabbit, monoclonal	-	1:1,000	Cell Signaling Technology, USA, Cat. #4257
SAPK/JNK, human	Rabbit, monoclonal	-	1:2,000	Cell Signaling Technology, USA, Cat. #9252
TRPM7, mouse	Mouse, monoclonal	1:100	1:500	Abcam, UK, Cat. #ab85016
Vimentin, human	Rabbit, monoclonal	1:200	1:2,000	Cell Signaling Technology, USA, Cat. #5741
Catalase, human	Rabbit, monoclonal	-	1:2,000	Abcam, UK, Cat. #ab76024
SOD2, human	Rabbit, monoclonal	-	1;1,000	Abcam, UK, Cat. #ab68155

**Table 3:** List of secondary antibodies and counterstaining of nuclei

Secondary detection system used	Host	Method	Dilution	Supplier
Anti-Mouse-IgG (H+L)-HRP	Goat	WB	1:10,000	Sungene Biotech, China, Cat. #LK2003
Anti-Rabbit-IgG (H+L)-HRP	Goat	WB	1:10,000	Sungene Biotech, China, Cat. #LK2001
Anti-rabbit IgG (H+L), F(ab')2 Fragment (Alexa Fluor® 488 Conjugate)	Goat	IF	1:50	Cell Signaling Technology, USA, Cat. #4412
Anti-rabbit IgG (H+L), F(ab')2 Fragment (Alexa Fluor® 555 Conjugate)	Goat	IF	1:50	Cell Signaling Technology, USA, Cat. #4413
Anti-mouse IgG (H+L), F(ab')2 Fragment (Alexa Fluor® 488 Conjugate)	Goat	IF	1:50	Cell Signaling Technology, USA, Cat. #4407
Anti-mouse IgG (H+L), F(ab')2 Fragment (Alexa Fluor® 555 Conjugate)	Goat	IF	1:50	Cell Signaling Technology, USA, Cat. #4408
Hoechst 33342 nucleic acid staining (DAPI)	-	IF	1:750	Molecular Probes/Invitrogen, Carlsbad, CA, USA, Cat. #A11007
TOTO-3 iodide	-	IF	1:750	Molecular Probes/Invitrogen, Carlsbad, CA, USA, Cat. #T3604

### Immunofluorescence staining for human bladder tissue samples

Histological diagnosis of bladder tissues were examined by two experienced pathologists independently. All the samples were fixed by 4% PFA at 4°C overnight and embedded into paraffin (Paraplast, Sigma-Aldrich) using tissue processor (Thermo Fisher Scientific, Cat. #STP 120). Paraffin sections (4 μm) were cut with a rotation microtome (Thermo Fisher Scientific, Cat. #HM325). The sections were serially incubated with indicated primary antibody (listed in Table 2) and Cy3-labeled or FITC- labeled secondary antibody (listed in Table 3) in humidified atmosphere. Nuclei were labeled with DAPI (2 μg/ml). Sections were analyzed by a fluorescence microscope (Olympus, Cat. #IX73).

### Immunofluorescence analysis for BCa cells

BCa cells were seeded on 12 mm coverslips, washed three times with ice cold PBS and fixed with 4% PFA for 30 min. Cells were then treated with 0.1% Triton X-100 solution and blocked in normal goat serum for 30 min at room temperature. Afterwards, cells were incubated with the indicated primary antibody (Table 2) at the proper dilution for 2 h at room temperature, washed three times with PBS, and incubated with Cy3-labeled or FITC- labeled secondary antibody (Table 3) for 1 h. Nuclei were visualized with 1 mM TOTO-3 iodide for 10 min at room temperature. Immunofluorescence staining was analyzed using a confocal microscope (Leica, Cat. #SP8).

### Xenograft model

Male NOD/SCID mice were purchased from Beijing HFK Bioscience Co., Ltd. in Beijing, China. Six mice at Day 42 were subcutaneously injected with T24 cells at a concentration of 4 × 10^7^/ml diluted in PBS (100 μl for each mouse) and grown for 14 days [75]. Three mice of them were intraperitoneally injected by carvacrol at 50 mg/ml (200 μl for each mouse) and repeated for 28 days to inhibit activity of TRPM7 [69]. The other three mice were treated by 0.9% saline (200 μl for each mouse) for 28 days as control.

*Lentiviral-TRPM7-shRNA* and *lentiviral-control-shRNA* infected T24 cells were subcutaneously injected to ten mice, respectively, diluted in PBS (200 μl per mouse) at a concentration of 2 × 10^7^/ml cells. The tumor size for each mouse was measured every four days using a caliper and counted as: tumor size = length × width^2^ × 0.5 mm^3^ [76]. The growth of the tumor was observed for 42 days, and the mice was sacrificed to measure the tumor weight.

### Statistical analyses

Data were expressed as mean ± SD from three independent experiments. All analyses were performed three times and represent data from three individual experiments. Two-tailed Student's *t*-tests and one-way analysis of variance (ANOVA) were used to evaluate the statistical significance of differences of the data. All of the statistical analyses were performed with SPSS16.0. The statistical significance was set at probability values of *p* < 0.05.

## SUPPLEMENTARY MATERIALS AND FIGURES








