# Supplementary material for: Decreased TRPM7 inhibits activities and induces apoptosis of bladder cancer cells via ERK1/2 pathway
Source: Oncotarget. 2016 Sep 20;7(45):72941–60. doi: 10.18632/oncotarget.12146 (PMC5341955; doi:10.18632/oncotarget.12146)
Supplement: Supplementary file 1 [file oncotarget-07-72941-s001.pdf]

Decreased *TRPM7* inhibits activities and induces apoptosis of bladder cancer cells via ERK1/2 pathway

Supplementary Materials

Supplementary Information S1: Approval by the Ethics Committee at Zhongnan Hospital of Wuhan University (approval number: 2015029) for the microarray and qRT-PCR analysis using RNA isolated from human bladder cancer tissues (stage II) and normal bladder epithelium from donors by accidental death. See Supplementary_ Information S1

Supplementary Information S2: Significantly altered 1338 genes (fold change > 1.5) revealed by microarray analysis for the mRNA isolated from three bladder cancer tissues versus three normal bladder tissues. The analysis was by Gene Cloud of Biotechnology Information software (GCBI System, China) (www.gcbi.com.cn) and the data was submitted to GEO database (accession number: GSE76211). See Supplementary_ Information S2

Supplementary Information S3: Significantly affected 146 signaling pathways revealed by microarray analysis comparing mRNA isolated from three bladder cancer tissues with three normal bladder tissues. Microarray analysis was using the GCBI analysis tool. See Supplementary_ Information S3

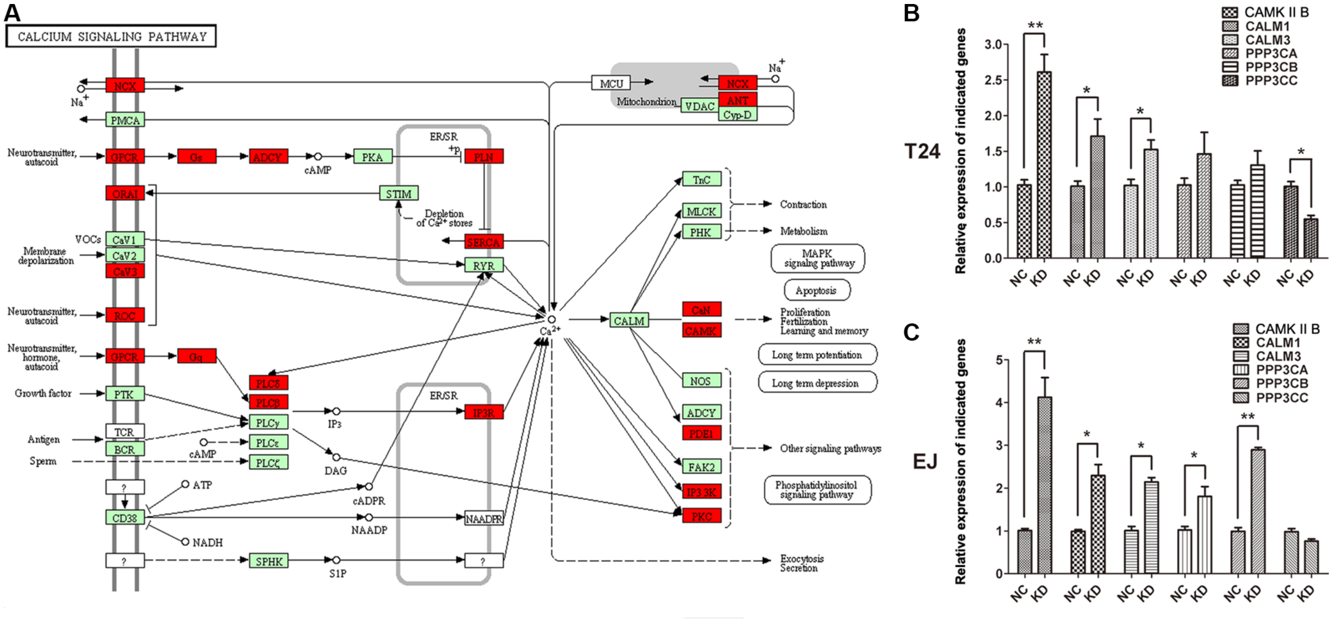

Supplementary Figure S1: Calcium signalling pathway was altered in the BCa tissues and *TRPM7* downregulated BCa cells. (**A**) Overrepresentation analysis using microarray raw data and DAVID database revealed altered genes (marked in red) involved in calcium signaling pathway. The calcium signaling pathway was modified from Kyoto Encyclopedia of Genes and Genomes (KEGG) Pathway Database. (**B–C**) qRT-PCR analysis for relative gene expression of *CAMK II B* (subtype of CAMK), *CALM1/3* (subtypes of CALM) and *PPP3CA/B/C* (subtypes of CaN) in the T24 (B) and EJ cells (C) with *TRPM7*-siRNA (KD) versus *control*-siRNA treatment (NC). Values of statistical data shown were mean ± SD of triplicate measurements and repeated three times with similar results. *$p < 0.05$, **$p < 0.01$.

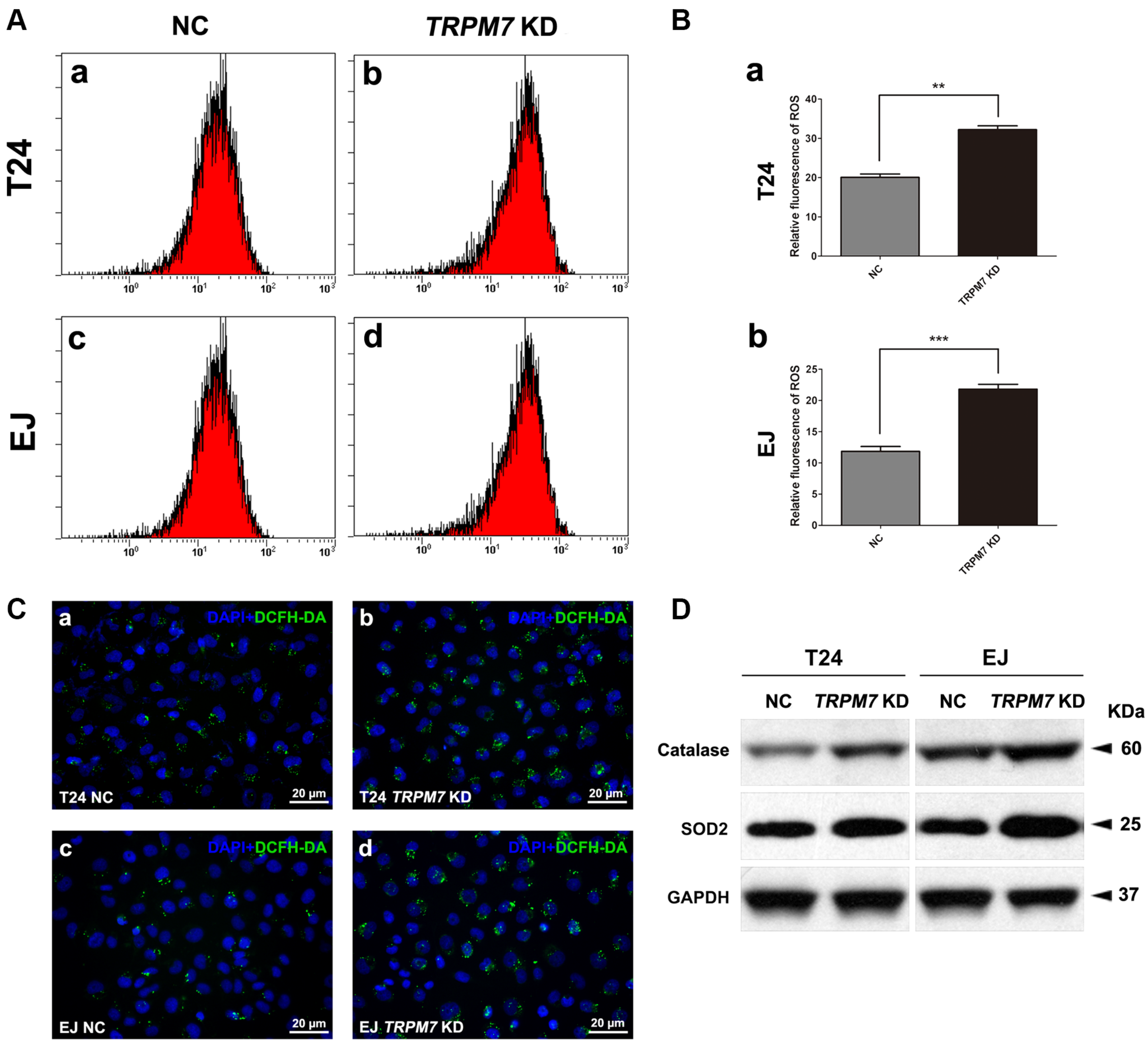

Supplementary Figure S2: Increased ROS status triggered by downregulated *TRPM7* in BCa cells. (**A**) Flow cytometry analysis for T24 (a-b) and EJ (c-d) cells stained with DCFH-DA, and statistically analyzed in (**B**), revealing an increased ROS in the T24 (a) and EJ (b) cells. (**C**) DCFH-DA stained (green) T24 (a-b) and EJ (c-d) cells after *TRPM7-siRNA* (*siTRPM7*) and control-siRNA (*siCON*) treatment. Nuclears were stained by DAPI (blue). The images were photographed by fluorescence microscope and scale bars for C are 20 µm. (**D**) Western blot analysis for proteins involved in ROS metabolism (Calatase and SOD2). GAPDH was used as a loading control (cell types, treatment of *siRNA* and protein masses were indicated).

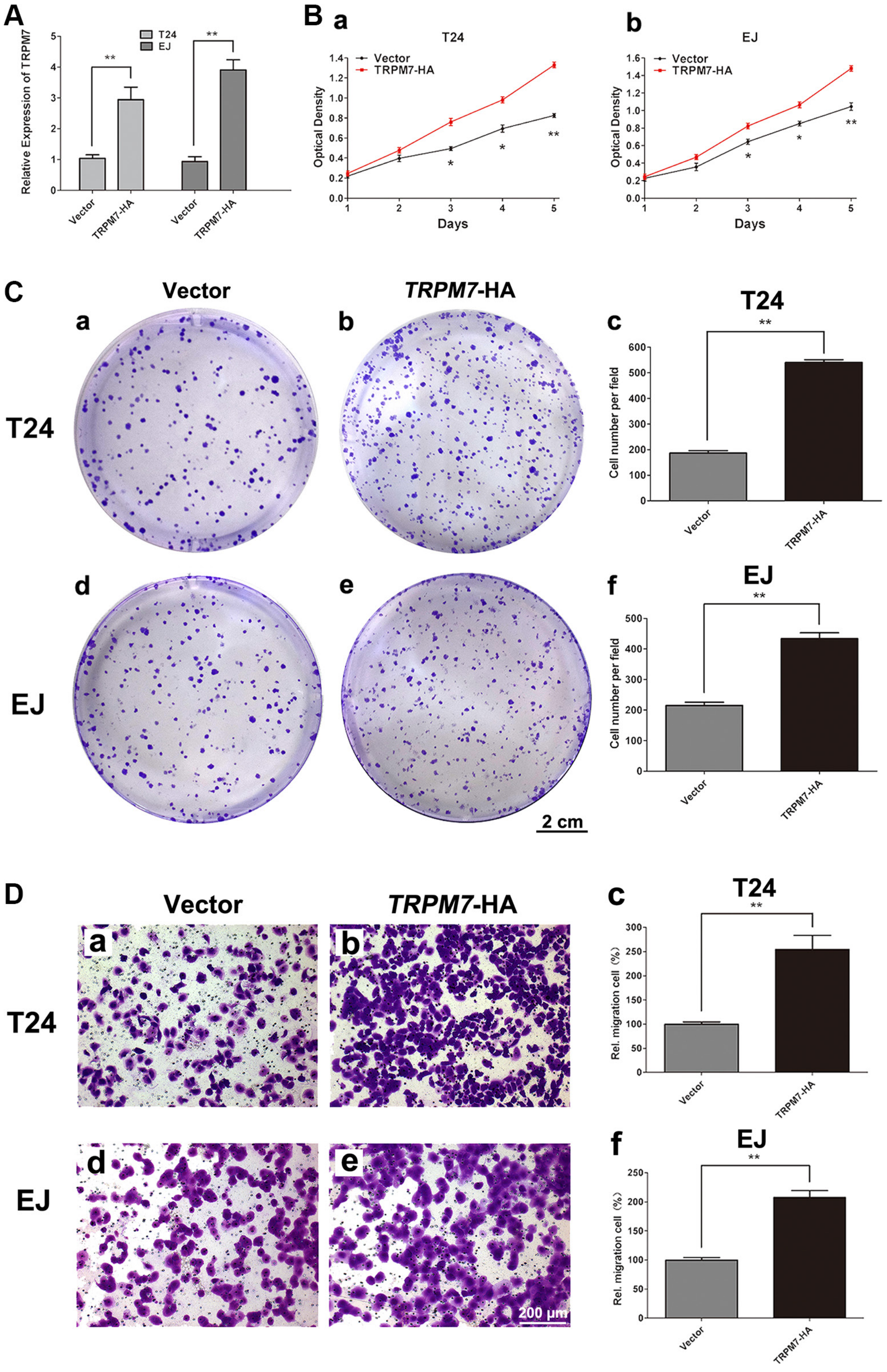

Supplementary Figure S3: *TRPM7*-overexpression induced BCa cell proliferation and motility. (**A**) qRT-PCR analysis for relative gene expression of *TRPM7* in the T24 and EJ cells transfected with pcDNA5/FRT/TO/HA-TRPM7 vector (*TRPM7*-HA) and control. $^{**}p < 0.01$. (**B**) CCK-8 assay for T24 (a) and EJ (b) proliferation (vector type, optical density and days after transfection were indicated). $^{*}p < 0.05$, $^{**}p < 0.01$. (**C**) Cell proliferation of BCa cells T24 (a-b) and EJ (d-e) were evaluated by clonogenic survival assay after transfection with *TRPM7*-overexpression vector (*TRPM7*-HA) and control vector. Statistical analysis revealed a significant increase of cell number per field in T24 (c) and EJ (f) cells. $^{**}p < 0.01$. The scale bar for C is 2 cm. (**D**) For transwell migration analysis the T24 (a- b) and EJ (d-e) cells were incubated in the upper transwell chambers for 24 h and the number of migrated cells was counted in five random fields per chamber by phase contrast microscope (a) and statistically analyzed (c and f). Cell type, Vector type and relative migration rate were indicated. All values shown were mean ± SD of triplicate measurements and repeated three times with similar results, $^{**}p < 0.01$. The scale bar for D is 200 μm.
