# Supplementary material for: Decreased TRPM7 inhibits activities and induces apoptosis of bladder cancer cells via ERK1/2 pathway
Source: Oncotarget. 2016 Sep 20;7(45):72941–60. doi: 10.18632/oncotarget.12146 (PMC5341955; doi:10.18632/oncotarget.12146)
Supplement: Supplementary file 2 [file oncotarget-07-72941-s002.pdf]

武汉大学中南医院医学伦理委员会科研项目伦理审批件

科伦[2015029]

<table>
<tr><td>项目名称</td><td colspan="6">泌尿系疾病发病机制及分子诊治的科学研究</td></tr>
<tr><td>申报单位</td><td colspan="6">武汉大学中南医院</td></tr>
<tr><td>项目负责人</td><td colspan="4">王行环</td><td>科室</td><td>泌尿外科</td></tr>
<tr><td>课题来源</td><td colspan="6">武汉市科技局</td></tr>
<tr><td>审查文件</td><td colspan="6">详见附件</td></tr>
<tr><td>审查类别</td><td>初始审查</td><td colspan="2">审查方式</td><td colspan="3">会议审查</td></tr>
<tr><td>审查日期</td><td>2015-07-29</td><td colspan="2">审查地点</td><td colspan="3">中南医院门诊 13 楼 2 号会议室</td></tr>
<tr><td>出席人员</td><td>实到 10 人</td><td colspan="3">回避 0 人</td><td colspan="2">弃权 0 人</td></tr>
<tr><td>投票结果</td><td>同意 3 票</td><td>作必要的修正后同意 7 票</td><td>作必要的修正后重审 0 票</td><td colspan="2">不同意 0 票</td><td>暂停或终止已经批准的试验 0 票</td></tr>
<tr><td>审查意见</td><td colspan="6">经本伦理委员会审查，审查决定如下：
□同意 ■作必要的修正后同意 □作必要的修正后重审
□不同意 □终止或暂停已批准的试验

主任委员或副主任委员签字：[illegible]
武汉大学中南医院医学伦理委员会（盖章）：
日期：2015-7.31</td></tr>
<tr><td colspan="7">注意事项：</td></tr>
<tr><td colspan="7">1. 请遵循 CFDA/GCP 原则和《赫尔辛基宣言》、遵循本伦理委员会批准的方案开展临床研究，保护受试者的健康与权利。
2. 研究过程中，对研究方案和知情同意书等相关文件的修改，均须得到伦理委员会审查同意后方可实施。
3. 发生严重不良事件或影响研究风险受益比的非预期不良事件、违背方案、暂停/提前终止研究须及时报告本伦理委员会。
4. 根据项目来源类别提交跟踪审查材料。</td></tr>
</table>

伦理委员会声明：本伦理委员会严格按照中国 GCP 及相关法律法规组成及工作。

伦理委员会地址：湖北省武汉市武昌区东湖路 169 号；邮编 430071；电话：027-67812787。

复 审 表

项　目	泌尿系疾病发病机制及分子诊治的科学研究		
申办者			
方案版本号		方案版本日期	
知情同意书版本号		知情同意书版本日期	
研究专业/PI	王行环/泌尿外科		

审查意见 ：

1.所做修改是否符合上次审查意见

☑是　□否

2.是否需要进一步修正

□是　☑否

3.是否提交会议审查

□是　☑否

主审委员审查意见	☑同意　□作必要的修正后同意　□作必要的修正后重审<br>□不同意　□终止或暂停已批准的试验<br>建议：________<br>主审委员签名：[illegible]　日期：2016.2.3

医学伦理委员会评审结果：

☑同意　□作必要的修正后同意　□作必要的修正后重审

□不同意　□终止或暂停已批准的试验

主任委员或副主任委员签名/日期	[illegible] 2016.2.3

武汉大学中南医院医学伦理委员会审查资料目录

项目名称：泌尿系疾病发病机制及分子诊治的科学研究

1. 伦理审查申请表
2. 项目申请书
3. 质量管理方案
4. 伦理流程
5. 知情同意书
6. 湖北省人体器官捐献登记表
7. 证明书
8. 研究者简历
